# Blocking of Transient Receptor Potential Vanilloid 1 (TRPV1) promotes terminal mitophagy in multiple myeloma, disturbing calcium homeostasis and targeting ubiquitin pathway and bortezomib-induced unfolded protein response

**DOI:** 10.1186/s13045-020-00993-0

**Published:** 2020-11-25

**Authors:** Katia Beider, Evgenia Rosenberg, Valeria Dimenshtein-Voevoda, Yaarit Sirovsky, Julia Vladimirsky, Hila Magen, Olga Ostrovsky, Avichai Shimoni, Zohar Bromberg, Lola Weiss, Amnon Peled, Arnon Nagler

**Affiliations:** 1 Division of Hematology, CBB and Bone Marrow Transplantation, Chaim Sheba Medical Center, Tel Aviv University, Tel-Hashomer, Ramat Gan, Israel; 2 Goldyne Savad Institute of Gene Therapy, Hebrew University Hospital, Jerusalem, Israel

**Keywords:** Multiple myeloma, Bortezomib resistance, TRPV1, Calcium signaling, Mitochondrial UPR

## Abstract

**Background:**

Chemoresistance remains a major treatment obstacle in multiple myeloma (MM). Novel new therapies are thus in need. Transient Receptor Potential Vanilloid type 1 (TRPV1) is a calcium-permeable ion channel that has been demonstrated to be expressed in solid tumors. Calcium channels have been shown to be involved in the regulation of cell proliferation, chemoresistance, migration and invasion. The aim of the current study was to evaluate its possible role in MM.

**Methods:**

Pharmacological inhibitor was used to evaluate the role of TRPV1 in MM cell lines and primary MM cells. Flow cytometry, molecular analysis, fluorescent microscopy, proteomic analysis and xenograft in vivo model of MM with BM involvement were employed to assess the effect of TRPV1 inhibition and decipher its unique mechanism of action in MM.

**Results:**

TRPV1 was found to be expressed by MM cell lines and primary MM cells. TRPV1 inhibition using the antagonist AMG9810-induced MM cell apoptosis and synergized with bortezomib, overcoming both CXCR4-dependent stroma-mediated and acquired resistance. In accordance, AMG9810 suppressed the expression and activation of CXCR4 in MM cells. TRPV1 inhibition increased mitochondrial calcium levels with subsequent mitochondrial ROS accumulation and depolarization. These effects were reversed by calcium chelation, suggesting the role of calcium perturbations in oxidative stress and mitochondrial destabilization. Furthermore, AMG9810 abolished bortezomib-induced accumulation of mitochondrial HSP70 and suppressed protective mitochondrial unfolded protein response. Proteomics revealed unique molecular signature related to the modification of ubiquitin signaling pathway. Consequently, 38 proteins related to the ubiquitylation machinery were downregulated upon combined bortezomib/AMG9810 treatment. Concomitantly, AMG9810 abolished bortezomib-induced ubiquitination of cytosolic and mitochondrial proteins. Furthermore, bortezomib/AMG9810 treatment induced mitochondrial accumulation of PINK1, significantly reduced the mitochondrial mass and promoted mitochondrial-lysosomal fusion, indicating massive mitophagy. Finally, in a recently developed xenograft model of systemic MM with BM involvement, bortezomib/AMG9810 treatment effectively reduced tumor burden in the BM of MM-bearing mice.

**Conclusions:**

Altogether, our results unravel the mechanism mediating the strong synergistic anti-MM activity of bortezomib in combination with TRPV1 inhibition which may be translated into the clinic.

## Introduction

Multiple myeloma (MM) is a neoplastic disorder that is characterized by clonal proliferation of plasma cells in the bone marrow (BM). It accounts for 10% of all hematological malignancies [1, 2]. Development of novel pharmaceutical agents has resulted in major advances in the treatment of MM in the last two decades [3]. Treatment strategies with that combined immune-modulatory drugs, proteasome inhibitors, conventional chemotherapy and monoclonal antibodies resulted in substantial progression in treatment outcome. However, despite improvements in patient survival rates, MM remains an incurable disease. Acquired or de novo resistance to current anti-MM therapy remains a major treatment obstacle [4, 5]. Novel new therapies are thus in need.

The transient receptor potential (TRP) channel superfamily is one of the largest families of cation channels [6]. Transient Receptor Potential Vanilloid type 1 (TRPV1) was the first identified and is the most extensively studied member of the vanilloid receptor subfamily of TRP ion channels. The TRPV1 receptor is a non-selective cation channel with a preference for calcium transmission and was initially identified as a specific receptor for capsaicin that causes a burning sensation [7]. Further studies revealed that TRPV1 is a polymodal receptor and is sensitive to multiple external stimuli that include capsaicin, ethanol, temperature above 43 °C and acid/basic pH changes [8, 9]. TRPV1 channel is involved in the regulation of calcium signaling, crucial for many cellular processes including proliferation, apoptosis, secretion of cytokines or T cell activation [10, 11]. Its broad expression in a wide range of tissues such as skin, respiratory airways, gastrointestinal tract, urinary epithelial cells, pancreatic B cells and he immune system, underlines the important role of TRPV1 [12–17]. Currently, TRPV1 was shown to be implicated in neurogenic inflammation, neuropathic pain, autoimmune disorders, immune cells functioning and cancer [11]. Functional expression of TRPV1 was demonstrated in several human malignancies including breast, prostate, urothelial cancer and glioma [18–22].

The role of TRPV1 channel in MM tumor progression and drug resistance has not been studied. We, therefore, evaluated the role of TRPV1 channel in MM, demonstrating that TRPV1 is expressed in MM cell lines and primary MM cells. TRPV1 inhibition using pharmacological blocker AMG9810 promotes MM cell death and synergizes with anti-MM agent bortezomib. Furthermore, our results reveal the mechanism that underlies the synergism between bortezomib and AMG9810, demonstrating that TRPV1 inhibition disturbs mitochondrial calcium signaling, suppresses bortezomib-induced mitochondrial unfolded protein response (mtUPR) and promotes mitophagy. Altogether, simultaneous TRPV1 and proteasome targeting appears to be promising novel therapeutic strategy in MM.

## Materials and methods

### Cell lines and MM patient samples

The following human MM cell lines were obtained from ATCC (Rockville, MD, USA): RPMI8226, U266 and NCI-H929. The CAG MM cell line (generated by the group at the University of Arkansas for Medical Sciences (UAMS) [23]), OPM-1 and OPM-2 (originate from the same individual) were kindly donated by Prof. Israel Vlodavsky, Technion, Israel. Cells were maintained in log-phase growth in RPMI1640 medium (Biological Industries, Israel) supplemented with 10% heat-inactivated fetal calf serum (FCS), 1 mM L-glutamine, 100 U/ml penicillin and 0.01 mg/ml streptomycin (Biological Industries) in a humidified atmosphere of 5% CO_2_ at 37 °C. MM cell lines were authenticated in 2019 at the Genomics Center of Biomedical Core Facility, Technion, using the Promega GenePrint 24 System. Primary MM cells were isolated from bone marrow aspirates of myeloma patients. The study was approved by Institutional Review Board of the Sheba Medical Center. Mononuclear cells were collected after separation on Ficoll-Paque (Pharmacia Biotech). MM cells were purified (> 95% purity) by CD138+ isolation using MACS magnetic cell sorter (Miltenyi Biotec Inc.).

### Preparation of BMSCs and co-culture experiments

Primary human bone marrow stromal cells (BMSCs) were generated from bone marrow aspirates of consenting healthy donor volunteers. BMSCs were isolated by plate adherence and expanded as previously described [24].

### Inhibitors

The following chemicals were used: bortezomib, carfilzomib, AMG9810, capsaicin and MLN4924 from Cayman.

### Cell line transduction

In order to stably over-express CXCR4, RPMI8226 cells were transduced with the lentiviral bicistronic vector encoding for CXCR4 and GFP genes, as previously described [25]. For CXCR4 silencing, cells with exogenously expressed CXCR4 (RPMI8226-CXCR4) were stably transduced with lentiviral vectors encoding for specific anti-CXCR4 short hairpin RNA (shRNA) (pLKO.1-shRNA-CXCR4 TRCN, Mission TRC Sigma) using the same envelope and packaging constructs.

### Analysis of surface markers

Expression levels of CXCR4 were evaluated by immune staining with allophycocyanin (APC)-conjugated anti-CXCR4 monoclonal antibody (12G5 clone) (eBioscience, USA). In primary MM samples, counterstaining with anti-CD138 fluorescein (FITC)-conjugated antibody (IQ products, Netherlands) was performed. The cells were analyzed by Navios (Becton Coulter), using Kaluza software.

### XTT viability assay

Cells were exposed in vitro to increasing concentrations of AMG9810 for 48 h, and viability was determined as described in Additional file 1.

### Cell cycle analysis

Cells were exposed in vitro to increasing concentrations of AMG9810 and bortezomib for 48 h and analyzed by FACS as described in Additional file 1.

### Assessment of apoptosis

Apoptosis was determined as described in Additional file 1.

### Quantitative RT-PCR analysis

RNA isolation and qRT-PCR analysis were performed as described in Additional file 1.

### Acridine Orange staining

To assess vesicle acidification, MM cells were exposed to AMG9810 (10 µM), bortezomib, capsaicin or their combinations for different time points and were loaded with 1 µg/ml acridine orange (AO) (Sigma), for 30 min in 37 °C, and analyzed by flow cytometry.

### Mitochondrial ROS accumulation

Following treatment with indicated reagents, levels of mitochondrial ROS were assessed using MitoSOX Red in combination with Annexin V-CF647 using FlowCellect™ MitoStress Kit (Merck Millipore) according to the manufacturer’s instructions.

### Mitochondrial mass detection

Mitochondrial mass in live cells treated with indicated reagents was monitored using the probe MitoSpy™ Red CMXRos (Biolegend) at final concentration of 100 nM, according to the manufacturer’s instructions, and analyzed by flow cytometry.

### Assessment of mitochondrial membrane potential (Δ*Ψm*)

Effect of AMG9810 and bortezomib treatment on Δ*Ψm* was evaluated using DiOC6 (Sigma-Aldrich) staining as previously described [24].

### Cell migration assay

Migration of MM cell lines in response to various CXCL12 concentrations (5–500 ng/ml) (PeproTech EC) was evaluated using 5-µm pore size transwells (Costar). The quantity of cells migrating within four hours to the lower compartment was determined by FACS and expressed as a percentage of the input. For TRPV1 inhibition, cells were pre-treated (30 min in 37 °C) with AMG9810 (10 µM) and subjected to migration.

### Cell adhesion assay

Cell adhesion was determined as described in Additional file 1.

### Assessment of lysosomal membrane permeabilization

MM cells were exposed to AMG9810 (5–10 µM), bortezomib (3–5 nM) or their combination for 24 or 48 h, labeled with LysoTracker (Cell Signaling Technology) for 30 min, 37 °C for 30 min, and analyzed by flow cytometry.

### Immunofluorescent staining and microscopy

MM cells were exposed to AMG9810 (10 µM), in the absence or presence of BAPTA-AM (5 µM) for 1 h. Next the cells were seeded on poly-D-lysine pre-coated slides for 30 min and loaded with Rhod-2 calcium marker (Invitrogen) for additional 30 min. Next, the cells were fixed with 100% ice-cold methanol for 5 min, washed with PBSx1 and permeabilized with 0.5% saponin for 30 min. After blocking non-specific binding with 1% BSA for 1 h, anti-COX IV antibody (1:500) (Cell Signaling Technology) in 0.5% saponin-containing buffer was applied for 2-h incubation. Thereafter, the slides were washed with PBSx1 and incubated with secondary anti-rabbit (1:500), FITC-conjugated antibody for 1 h and subsequently counterstained with DAPI-containing mounting solution (Vector Laboratories). Stained cells and negative controls were evaluated using an Olympus BX53 microscope connected to an Olympus DP73 camera (Olympus, Melville, NY, USA). Images were captured for analysis using cellSens imaging software (Olympus, Melville, NY, USA).

### Mitochondrial calcium and total cellular calcium measurements by flow cytometry

The mitochondrial calcium indicator Rhod-2, AM (Invitrogen), was used to assess mitochondrial calcium in MM cells. To evaluate total intracellular calcium levels, eFluor 514 (eBioscience™) calcium sensor dye was utilized. Cells were pre-treated with indicated treatments and then loaded with 10 μM Rhod-2 or 5 μM eFluor 514 for 30 min, washed with PBSx1 and analyzed by Navios (Beckman Coulter), using Kaluza software.

### Immunoblot analysis

Mitochondria/cytosol fractionation was performed using commercial kit (Biovision) according to the manufacturer’s instructions. Total protein lysates (50–70 μg) or mitochondria/cytosol fractions (30 μg) were resolved by electrophoresis in 10% SDS-PAGE and transferred onto PVDF membranes. Blots were subjected to a standard immunodetection procedure using specific antibodies and the ECL substrate (Biological Industries). Signal was detected using a Bio-Rad image analyzer (Bio-Rad). The primary antibodies used were: CHOP, BCL-2, MCL-1, BCL-XL, phospho-Erk1/2, phospho-AKT, phospho-pS6, HSP70, HSP40, COX IV, AIF, PINK1, VDAC, LAMP1, ubiquitin, α-tubulin (Cell Signaling Technology), MCL-1 (Santa-Cruz) and β-actin (Sigma-Aldrich).

### Mass spectrometry-based proteomics

RPMI8226 cells were treated with bortezomib (10 nM), AMG9810 (10 µM) or combination of both drugs and subjected to proteomic analysis (Smoler proteomics center, Technion). Briefly, the samples were digested by trypsin and analyzed by LC–MS/MS on Q Exactive plus (Thermo). The data were analyzed with MaxQuant 1.6.0.16 vs the Human Uniprot database. The identifications are filtered for proteins identified with false discovery rate (FDR) < 0.01 with at least 2 peptides in the project.

### Murine xenograft models of disseminated human MM and drug treatment

NSG mice were maintained under defined flora conditions at the Hebrew University Pathogen-Free Animal Facility (Jerusalem, Israel). All experiments were approved by the Animal Care Committee of the Hebrew University. Mice were injected intravenously with RPMI8226-CXCR4 human cells (5 × 10^6^/mouse). Endpoints were paraplegia and weight loss > 10%. The mice were killed on the same day the endpoint was reached. Disease was verified by measurement of human immunoglobulin in plasma of inoculated mice using the ELISA kit (Immunology Consultants Laboratory). To investigate the therapeutic potential of AMG9810 as a single agent or in combination with bortezomib, three days after inoculation with RPMI8226-CXCR4 cells, mice were randomized and treated with intraperitoneal (i.p.) injections of either AMG9810 (10 mg/kg) twice per week, subcutaneous injections of bortezomib (0.5 mg/kg) twice per week, or with a combination of both agents, for a total of 6 injections. Animals were killed 24 days after tumor inoculation.

### Statistical analyses

Data are expressed as the mean ± standard deviation (SD) or standard error (SE). Statistical comparisons of means were performed by a two-tailed unpaired Student's *t* test or the Mann–Whitney *U* test.

## Results

### The expression of TRPV1 is increased in MM cell lines and primary MM cells, while TRPV1 inhibition using AMG9810 suppresses MM cell viability

**Fig. 1:**
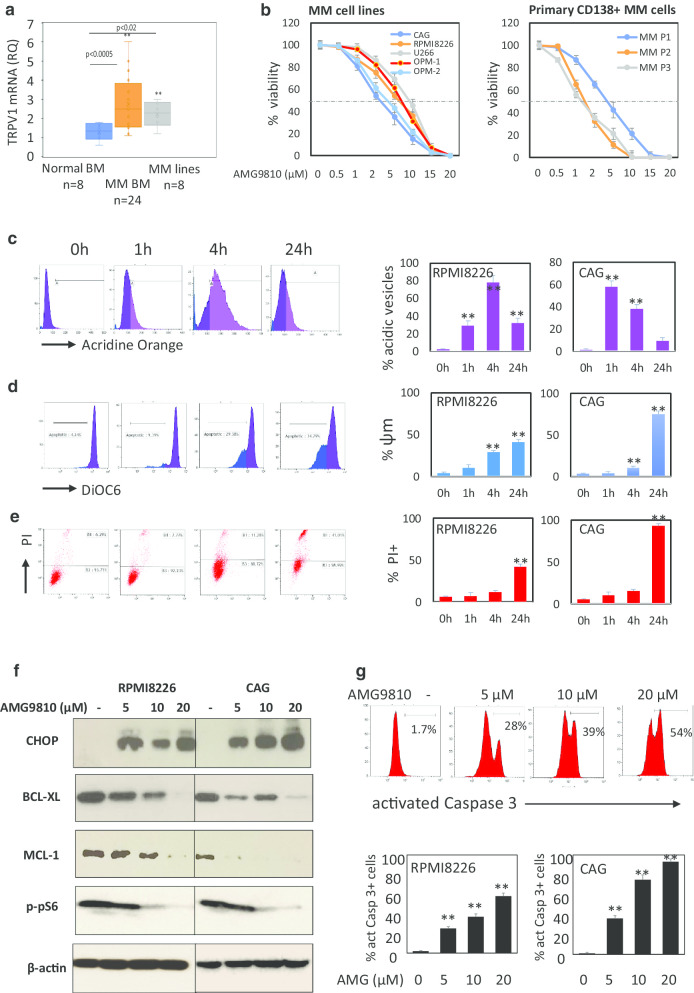
TRPV1 expression and the effect of TRPV1 inhibition on MM cell viability in MM BM samples and MM cell lines. **a** TRPV1 mRNA levels in bone marrow (BM) samples from healthy donors, MM patients and MM cell lines, measured by quantitative RT-PCR. **b** Effect of TRPV1 antagonist AMG9810 on viability of MM cell lines and primary CD138+ cells. Cells were incubated with increasing doses of AMG9810 for 48 h, and viability was measured by the XTT method. **c**–**e** Time course of the increase in AMG9810-induced vesicle acidification, mitochondrial depolarization and cell death. RPMI8226 and CAG MM cells were exposed to AMG9810 (10 µM) for indicated time points. **c** Vesicle acidification was measured by flow cytometry using acridine orange (AO) dye. Representative flow cytometry histograms from RPMI8226 cells (left side). Graphs showing percentages of AO-positive RPMI8226 and CAG cells obtained from representative experiment out of three repeats (right side). **d** Mitochondrial depolarization (Δ*Ψm* loss) in AMG9810-treated cells detected by DiOC6 staining and flow cytometry analysis. Representative flow cytometry histograms from RPMI8226 cells (left side) and graphs showing percentages of MM cells with Δ*Ψm* loss obtained from representative experiment out of three repeats (right side). **e** AMG9810-induced cell death detected by PI staining. Representative flow cytometry histograms from RPMI8226 cells (left side) and graphs showing the percentages of PI-positive dead RPMI8226 and CAG cells from representative experiment out of three repeats (right side). Data are presented as mean of triplicates ± SD (**p < 0.01). **f** Western blot analysis of anti-apoptotic proteins BCL-XL and MCL-1, ER stress marker CHOP and mTOR pathway target pS6 in RPMI826 and CAG MM cells before and after treatment with various doses of AMG9810 for 24 h. β-Actin was used as internal control. Representative data from at least two independent experiments are shown. **g** Caspase 3 activation in response to AMG9810 treatment (5–20 µM, 24-h exposure) was assessed using CaspGLOW Red staining and flow cytometry analysis. Representative histograms (upper panel) and graphs showing percentages of cells with activated Caspase 3 (lower panel). Data are presented as mean of triplicates ± SD (**p < 0.01)

In order to determine the role of TRPV1 in MM, we first evaluated TRPV1 expression in MM cell lines (*n* = 8) and BM samples from patients with MM (*n* = 24). Significantly higher levels of TRPV1 transcript were detected in MM BM primary samples and MM cell lines, comparing to normal BM samples (*n* = 8) (Fig. 1a). To determine the potential of TRPV1 inhibition in myeloma, we exposed a panel of MM cell lines and primary CD138 + MM cells to AMG9810, a specific inhibitor of TRPV1, for 48 h. Treatment with AMG9810 induced potent dose-dependent anti-proliferative effect, with IC_50_ ranging 2.5–9.5 µM for MM cell lines and 1.5–5 µM for primary MM cells (Fig. 1b). Assessment of apoptotic cell death using Annexin V/PI staining revealed significant increase in early (Annexin V+/PI−) and late (Annexin V+/PI+) apoptosis in MM cells. Importantly, treatment with AMG9810 did not affect cell viability in normal PBMCs (Additional file 1: Fig. 1). Further evaluation of cell death mechanisms triggered by AMG9810 revealed that TRPV1 inhibition using AMG9810 induces fast (1-h exposure) and massive accumulation of intracellular acidic vesicles (detected by acidic vesicle tracer acridine orange) that precedes the subsequent mitochondrial permeabilization (4 and 24 h of treatment) followed by cell death (24 h) (Fig. 1c, d). This was accompanied by elevation of C/EBP homologous protein (CHOP) protein level, indicating that AMG9810 induces profound endoplasmic reticulum (ER) stress. Furthermore, AMG9810 treatment dose-dependently decreased the levels of the anti-apoptotic proteins BCL-2 and MCL-1 and the phosphorylation of mTOR target, pro-survival factor pS6, in MM cells (Fig. 1e). Finally, we observed significant activation of executioner caspase 3 upon AMG9810 treatment (Fig. 1f). Altogether, these results suggest that TRPV1 inhibition with AMG9810 induces profound cytotoxicity in MM cells that is associated with mitochondrial destabilization and can involve both apoptosis and autophagy signaling pathways.

### TRPV1 inhibition using AMG9810 induces mitochondrial calcium flux and promotes mitochondrial ROS accumulation

**Fig. 2:**
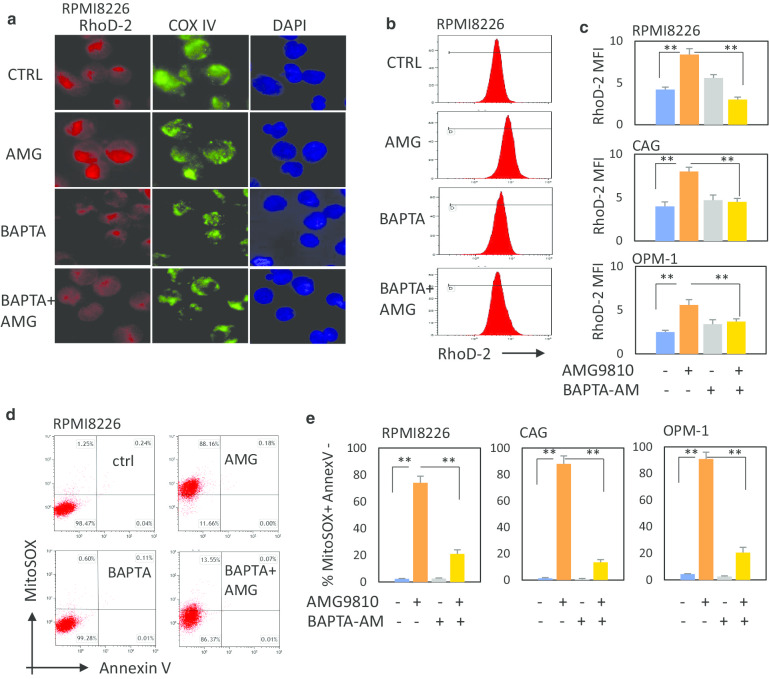
TRPV1 inhibition using AMG9810 induces increase in mitochondrial calcium levels and mitochondrial ROS levels in MM cells. MM cells were treated with AMG9810 (10 µM), with or without BAPTA-AM (5 µM) for 1 h, and mitochondrial calcium and mitochondrial ROS levels were evaluated. **a** RPMI8226 cells were labeled with fluorescent calcium indicator Rhod-2 (red fluorescence). To ensure the localization of mitochondria, cells were stained for mitochondrial marker COX IV (green fluorescence). The nucleus was stained with DAPI (blue fluorescence). Representative microscopy images show mitochondrial calcium, × 40 magnification. **b, c** RPMI8226, CAG and OPM-1 cells were treated with AMG9810 (10 µM), with or without BAPTA-AM (5 µM) for 1 h, stained with Rhod-2 and analyzed by flow cytometry. **b** Representative plots showing mitochondrial calcium change in response to various treatments, using the mean fluorescent intensity (MFI) of RhoD2 staining in RPMI8226 cells. **c** Mitochondrial calcium level in RPMI8226, CAG and OPM1 cells is presented as MFI of Rhod-2-stained cells. Data are presented as mean of triplicates ± SD (**p < 0.01). **d**, **e** Mitochondrial ROS levels and apoptosis were measured using MitoSOX and Annexin V staining. **d** Representative images showing MitoSOX/Annexin V staining in RPMI8226 cells (**e**)

Calcium is known to be a key regulator of mitochondrial function and regulates several processes including ATP synthesis and reactive oxygen species (ROS) generation [26]. Therefore, we next evaluated the effect of TRPV1 inhibition on mitochondrial calcium levels. MM cells (RPMI8226, CAG and OPM-1) were for 1 h exposed to AMG9810, labeled with mitochondrial calcium indicator RhoD-2 and analyzed by microscope and flow cytometer. To confirm the mitochondrial localization of RhoD-2 staining, MM cells were co-stained for mitochondrial marker COX IV (Fig. 2a). As demonstrated in Fig. 2a–c, blocking TRPV1 with AMG9810 resulted in an accumulation of mitochondrial calcium. To test whether the increase in RhoD-2 staining upon TRPV1 inhibition is dependent on calcium accumulation, cell-permeable calcium chelator BAPTA-AM was used. Pre-treatment with BAPTA-AM completely abolished the effect of AMG9810 and diminished the AMG9820-induced increase in RhoD-2 staining (Fig. 2a–c).

Mitochondrial calcium overload has been shown to promote ROS generation in mitochondria [27]. Therefore, the effect of AMG9810 on mitochondrial ROS levels was evaluated. Inhibition of TRPV1 using AMG9810 (1-h exposure) significantly increased mitochondrial ROS, as demonstrated by MitoSOX staining. Consistently, pre-incubation with calcium chelator BAPTA-AM abrogated the increase in mitochondrial ROS induced by AMG9810, suggesting that altered mitochondrial calcium can be the cause rather than a consequence of enhanced oxidative stress observed upon TRPV1 inhibition (Fig. 2d, e). To discriminate apoptotic cells, Annexin V co-staining was utilized. As we determined above, no apoptotic induction was detected following 1-h treatment with AMG9810. Of note, short-term exposure to TRPV1 agonist capsaicin (30 min) had no significant effect on mitochondrial ROS levels, resulting in delayed mild increase in mitochondrial ROS upon 4-h treatment (Additional file 1: Fig. 3A). Furthermore, prolonged TRPV1 inhibition using AMG9810 significantly increased total cellular calcium levels. In contrast, treatment with capsaicin had no significant effect on intracellular calcium following 24-h incubation (Additional file 1: Fig. 3B). Collectively, these results indicate that TRPV1 can be implicated in the regulation of mitochondrial calcium levels and mitochondrial redox signaling. Furthermore, our data point out the differential effect of TRPV1 antagonist AMG9810 on the mitochondria, suggesting that mitochondrial calcium overload triggered TRPV1 inhibition may result in prolonged uncompensated disequilibrium of intracellular calcium homeostasis and subsequent damage.

### TRPV1 inhibition using AMG9810 suppresses CXCR4 expression and activity in MM cells

**Fig. 3:**
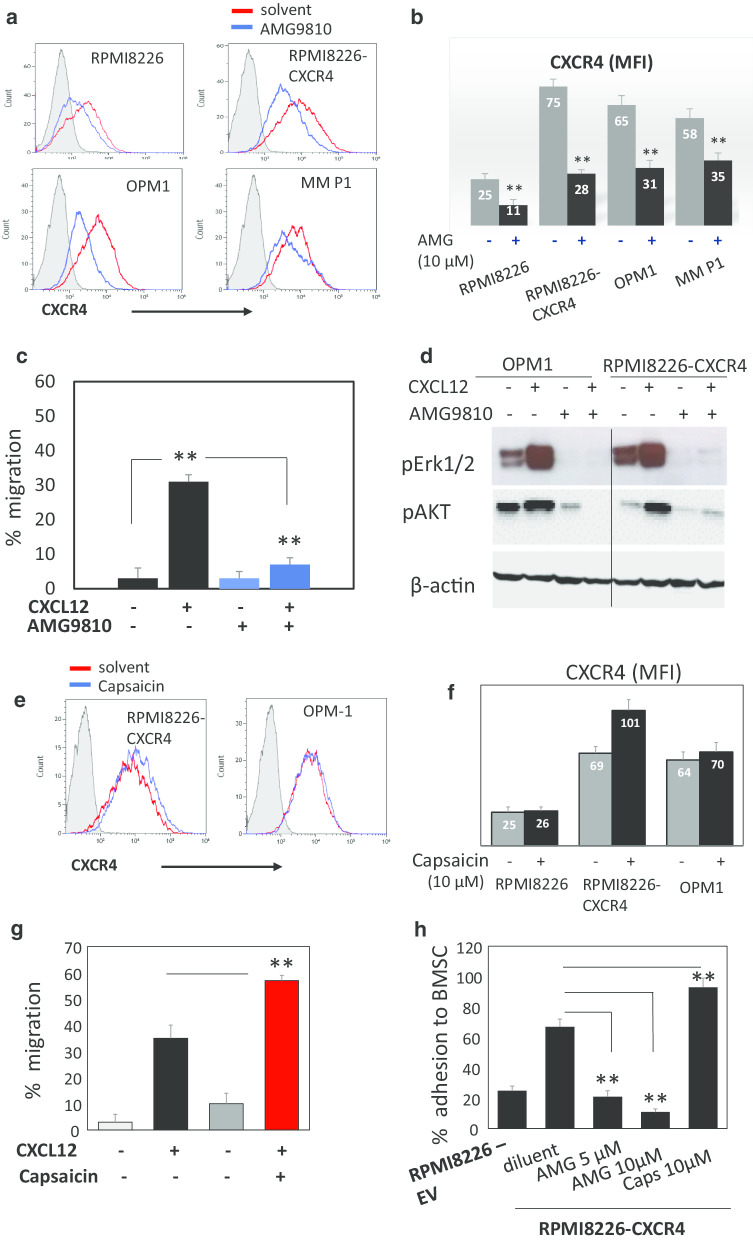
AMG9810 suppresses CXCR4 expression and activity in MM cells. Effect of AMG9810 treatment (10 µM, 1 h, 37 °C) on cell surface CXCR4 expression in RPMI8226, RPMI8226-CXCR4, OPM-1 and primary CD138 + MM cells, measured by flow cytometry. **a** Representative histograms demonstrating CXCR4 levels in non-treated and AMG9810-treated cells. **b** CXCR4 expression level is shown as mean fluorescent intensity (MFI), data are presented as mean of triplicates ± SD (**p < 0.01). **c** RMI8226-CXCR4 cells were pre-treated with indicated doses of AMG9810 for 30 min, and their migratory response to CXCL12 (200 ng/ml) was measured using the trans-well migration assay. **d** Effect of pre-treatment with AMG9810 (10 µM for 1 h at 37 °C) on CXCL12-induced (200 ng/ml, 30-min activation) intra-cellular signaling in RPMI8226-CXCR4 and OPM-1 cells. Levels of phosphorylated Erk1/2 and AKT proteins, measured by Western blot, β-actin was used as internal control. Representative data from at least two independent experiments are shown. **e**, **f** Effect of capsaicin treatment (10 µM, 1 h, 37 °C) on cell surface CXCR4 expression in RPMI8226, RPMI8226-CXCR4 and OPM-1 cells, measured by flow cytometry. **g** RMI8226-CXCR4 cells were pre-treated with indicated doses of capsaicin (10 µM) for 30 min, and their migratory response to CXCL12 (200 ng/ml) was measured using the trans-well migration assay. **h** CFSE-prelabeled RPMI8226 and RPMI8226-CXCR4 cells were pre-treated with AMG9810 (10 µM) or capsaicin (10 µM) for 30 min and then co-incubated with BMSC monolayer during 30 min; non-adherent cells were removed, and adherent CFSE-positive cells were enumerated using flow cytometry and expressed as percent of total input cells. Data are presented as mean of triplicates ± SD (**p < 0.01)

Calcium signaling is known to be tightly involved in the regulation of cancer cell migration and metastatic spread [28]. Furthermore, the role of TRPV1 channels in regulation of cell migration was previously demonstrated [29]. In MM, the CXCL12/CXCR4 chemokine axis plays a key role in MM cell migration and localization into protective BM niche [30]. Therefore, we assessed the effect of TRPV1 inhibition on CXCR4 expression and CXCR4-mediated signaling in MM cells. Treatment with AMG9810 significantly downregulated cell surface CXCR4 in MM cell lines with native (RPMI8226, OPM-1) and over-expressed CXCR4 (RPMI8226-CXCR4). Primary CD138 + MM cells responded similarly, and CXCR4 decrease in expression was detected in response to AMG9810 exposure (Fig. 3a, b). Furthermore, TRPV1 inhibition with AMG9810 completely abrogated the migratory response of CXCR4-expressing MM cells to CXCL12 chemokine (Fig. 3c). Accordingly, TRPV1 inhibition blocked CXCR4-dependent signaling, suppressing the phosphorylation of Erk1/2 and AKT kinases in response to CXCL12 stimulation in MM cells (Fig. 3d). In contrast, TRPV1 activation using the specific agonist capsaicin mildly upregulated cell surface CXCR4 (Fig. 3e, f) and significantly enhanced the migration of RPMI8226-CXCR4 cells in response to CXCL12 (Fig. 3g). Moreover, modulation of TRPV1 activity affected CXCR4-mediated adhesion of MM cells to the bone marrow stromal cells (BMSC). Thus, TRPV1 blockade using AMG9810 significantly suppressed the adhesion, while TRPV1 activation with capsaicin increased the percentage of adherent MM cells (Fig. 3h). Altogether, these data demonstrate that TRPV1 activity is involved in the regulation of CXCR4 expression and function in MM cells, while TRPV1 inhibition negatively regulates CXCR4 axis in MM.

### TRPV1 inhibitor AMG9810 increases the sensitivity to bortezomib and carfilzomib and overcomes bortezomib resistance in MM cells

**Fig. 4:**
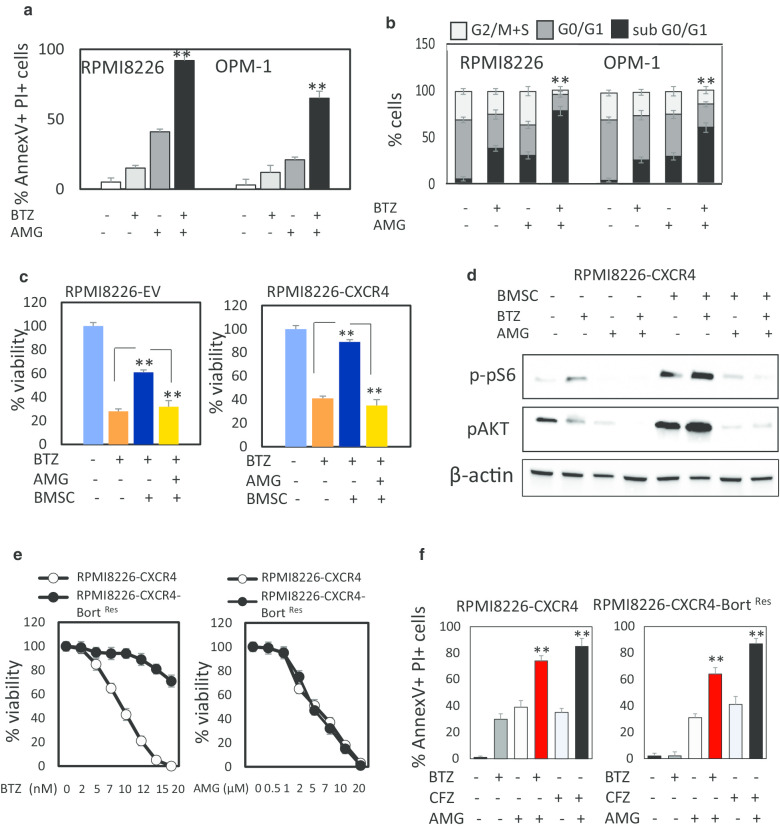
AMG9810 enhances the response to bortezomib in MM cells. **a**, **b** MM cells were exposed to low-dose bortezomib (BTZ) (3 nM) in the absence or presence of AMG9810 (10 µM) for 48 h. **a** Apoptosis was assessed using Annexin V/PI staining and flow cytometry analysis. Percentage of early (Annexin V+/PI−) and late (Annexin V+/PI+) apoptotic cells is presented as mean of triplicates ± SD (**p < 0.01). **b** Cell cycle distribution was determined using 7-AAD staining. Percent of proliferating cells (G2/M + S fractions), non-proliferating cells (G0/G1 fraction) and cells with fragmented DNA (sub G0/G1 fraction) are presented as mean of triplicates ± SD (**p < 0.01). **c** Effect of treatment with bortezomib (4 nM for RPMI8226-EV, 6 nM for RPMI8226-CXCR4), AMG9810 (10 µM) or their combination on the viability of MM cells incubated in the absence or presence of BMSCs, measured by PI-exclusion and flow cytometry analysis. **d** Effect of AMG9810 on BMSC-induced intra-cellular signaling in RPMI8226-CXCR4. RPMI8226-CXCR4 cells were treated with AMG9810 (10 µM), bortezomib (10 nM) or their combination in the absence or presence of BMSC for 24 h. Levels of phosphorylated AKT and pS6 proteins, measured by Western blot, β-actin were used as internal control. **e** RPMI8226-CXCR4 and RPMI8226-CXCR4-bortezomib-resistant cells were incubated during 48 h with indicated concentrations of bortezomib or AMG9810. Viability was evaluated using the XTT method. Results represent the average of triplicates ± SD (**p < 0.01). **f** RPMI8226-CXCR4 and RPMI8226-CXCR4-Bort^Res^ cells were exposed to bortezomib (BTZ) (10 nM) and carfilzomib (CFZ) (25 nM) in the absence or presence of AMG9810 (10 µM) for 48 h. Apoptosis was assessed using Annexin V/PI staining and flow cytometry analysis. Percentage of early (Annexin V+ /PI−) and late (Annexin V+/PI+) apoptotic cells is presented as mean of triplicates ± SD (**p < 0.01)

Bortezomib is commonly used in the triple first-line drug combinations for newly diagnosed MM patients. The development of bortezomib resistance is associated with major clinical problems during treatment of these patients. Therefore, exploring the mechanisms of resistance and strategies to overcome it is in need. Calcium signaling in cancer appears to have crucial role in cancer progression and drug resistance development, while targeting calcium pathway may represent a strategy for chemosensitization [31]. Here, we explored the effect of combination treatment with AMG9810 and bortezomib on MM cell viability. As depicted in Fig. 4a, combination of AMG9810 with bortezomib synergistically increased MM cells death. Moreover, combining AMG9810 with bortezomib interfered with cell cycle progression, markedly decreasing the proliferation, indicated by the percentage of cells in G2/M + S fractions and promoted apoptosis with DNA fragmentation, detected as increased subG0/G1 fraction (Fig. 4b). These results demonstrate that TRPV1 inhibition using AMG9810 enhances bortezomib-induced cytotoxicity, promoting apoptotic cell death with DNA fragmentation in MM cells.

Based on the ability of AMG9810 to suppress CXCR4 expression and activation on MM cells and given the role of CXCR4 in contact-dependent drug resistance (CAM-DR) of MM cells, we evaluated the effect of TRPV1 inhibition on stroma-mediated protection. Cells with native (RPMI822-EV) and over-expressed CXCR4 (RPMI8226-CXCR4, previously established in our laboratory [25]) were exposed to bortezomib in the absence or presence of BMSC. The presence of BMSC reduced the sensitivity of MM cells to bortezomib, providing partial protection to low CXCR4 expressing RPMI8226-EV cells and almost fully protecting the cells with high CXCR4. Importantly, addition of AMG9810 effectively reversed this stroma-mediated protection (Fig. 4c). To further elucidate the effect of TRPV1 inhibition on stroma-mediated resistance to bortezomib, we evaluated the activation of pro-survival signaling pathways implicated in CAM-DR. As shown in Fig. 4d, the presence of BMSC significantly increased the phosphorylation of AKT kinase and mTOR target pS6. However, TRPV1 inhibition using AMG9810, alone or in combination with bortezomib, effectively suppressed this stroma-mediated phosphorylation. As mentioned above, in order to explore the mechanisms of acquired resistance to bortezomib treatment, we previously established bortezomib-resistant cells by exposing RPMI8226-CXCR4 cells to gradually increasing concentrations of bortezomib. RPMI8226-CXCR4-Bort^Res^ cells displayed a significantly decreased sensitivity to the cytotoxic effect of bortezomib. However, AMG9810 treatment inhibited cell viability irrespective of bortezomib sensitivity, demonstrating a similar dose response in both bortezomib-sensitive and resistant lines, respectively (Fig. 4e). The combination of AMG9810 with bortezomib effectively targeted bortezomib-resistant MM cells and resulted in significantly increased cytotoxicity toward both RPMI8226-CXCR4 and RPMI8226-CXCR4-Bort ^Res^ cells. Moreover, TRPV1 inhibition was effective in combination with second-generation proteasome inhibitor carfilzomib, significantly increasing MM cell death (Fig. 4f). Based on these results we conclude that TRPV1 inhibition sensitizes MM to bortezomib and carfilzomib and effectively targets both CXCR4-dependent stroma-mediated as well as acquired resistance.

### Combining bortezomib with TRPV1 inhibitor AMG9810 promotes mitochondrial damage and induces lysosomal destabilization and uncompensated ER and oxidative stress in MM cells

**Fig. 5:**
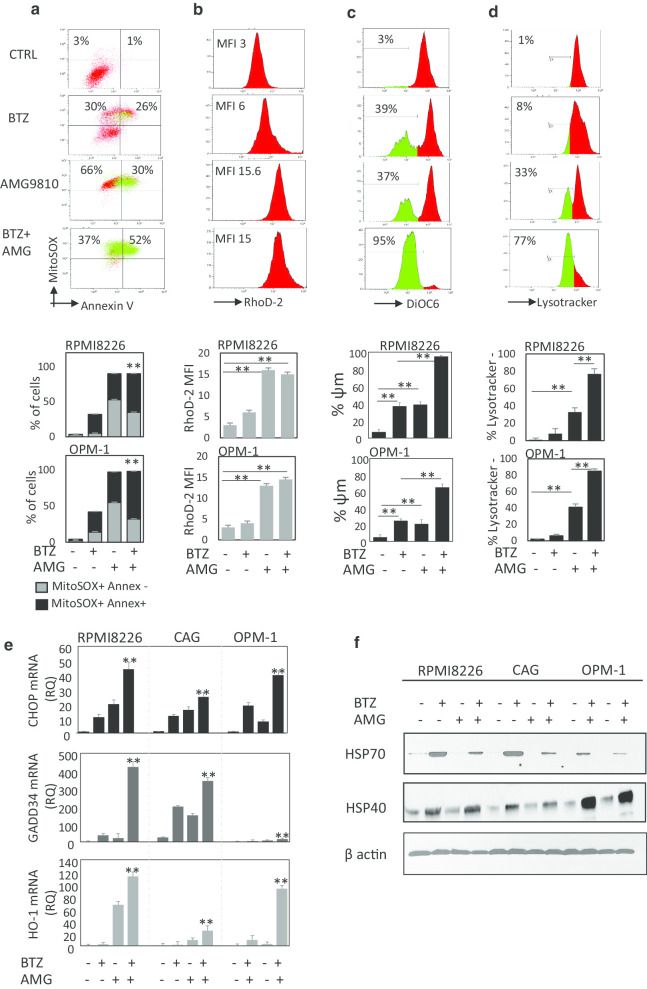
AMG9810 in combination with bortezomib elevates mitochondrial ROS and calcium levels, induces mitochondrial and lysosomal destabilization and promotes ER stress. RPMI8226 and OPM1 cells were exposed to low-dose bortezomib (BTZ) (4 nM) in the absence or presence of AMG9810 (10 µM) for 24 h. **a** Mitochondrial ROS levels and apoptosis were measured using MitoSOX and Annexin V staining. **b** Mitochondrial calcium levels were assessed using Rhod-2 staining. **c** Mitochondrial depolarization (Δ*Ψm* loss) was detected by DiOC6 staining and flow cytometry analysis. **d** Lysosomal membrane destabilization was assessed using LysoTracker staining. Representative images with RPMI8226 cells are shown in upper panels. Bars showing mean of triplicates ± SD (**p < 0.01) in RPMI8226 and OPM-1 cells are presented in lower panels. **e** RPMI8226, CAG and OPM-1 cells were treated with bortezomib (5 nM), AMG9810 (10 µM) or their combination for 24 h. RNA was extracted and was subjected to qRT-PCR analysis. CHOP, GADD34 and HO-1 mRNA levels were evaluated, using β2-microglobulin as endogenous control gene. **f** Western blot analysis of ER stress response chaperons HSP70 and HSP40 in RPMI826, CAG and OPM-1 cells before and after 24-h treatment with bortezomib (5 nM), AMG9810 (10 µM) or their combination. β-Actin was used as internal control. Representative data from at least two independent experiments are shown

To determine the molecular mechanisms responsible for the synergistic cytotoxic effect induced by combined bortezomib/AMG9810 treatment and taking into account central role of mitochondria in apoptosis induction, apoptosis-related molecular pathways were analyzed. First, we observed significant increase in mitochondrial ROS levels and associated apoptosis in bortezomib/AMG9810 MM-treated cells, as determined by MitoSOX/Annexin V-positive fraction (Fig. 5a). These changes were accompanied by profound increase in mitochondrial calcium levels, mainly promoted by TRPV1 inhibition (Fig. 5b). Furthermore, bortezomib/AMG9810 combination significantly decreased mitochondrial membrane potential (Δ*Ψm*), detected by decrease in DiOC6 staining (Fig. 5c). Importantly, combining bortezomib with AMG9810 induced profound lysosomal destabilization, as demonstrated by decrease in the LysoTracker staining (Fig. 5d). These results suggest that the increased cytotoxicity obtained upon combination treatment with bortezomib and AMG9810 may rely on the ability of TRPV1 antagonist to interfere with calcium signaling, inducing mitochondrial calcium flux, rapid increase in mitochondrial ROS production, followed by mitochondrial damage and lysosomal destabilization.

Excess cellular levels of ROS can be deleterious and trigger oxidative stress, causing oxidative damage to DNA and proteins and subsequent cell death [32]. Recent studies link between oxidative stress and ER stress and demonstrate that increased ROS may enhance the production of misfolded proteins, therefore inducing ER stress [33, 34]. In order to confirm it and to evaluate oxidative stress and ER stress induction in response to bortezomib/AMG9810 treatment, mRNA levels of CHOP and DNA damage-inducible 34 (GADD34) (ER stress response genes) as well as heme oxygenase-1 (HO-1) (oxidative stress response gene) were assessed by qRT-PCR. Accordingly, combined bortezomib/AMG9810 treatment resulted in significantly higher expression of ER stress and oxidative stress markers in comparison with a single agent exposure, reflecting enhanced ER stress in MM cells (Fig. 5e).

Induction of ER stress is one of the major mechanisms of bortezomib-mediated cell death. As a response to ER stress conditions, the unfolded protein response (UPR) signal cascade is activated to counteract the occurring damage. The UPR also induces the expression of heat-shock factor chaperons including HSP70 and HSP40 that play cytoprotective role enabling protein folding and reducing ER stress. Consistently, we detected significant increase in HSP70 and HSP40 protein levels in MM cells upon bortezomib treatment. However, TRPV1 inhibition using AMG9810 markedly decreased HSP70 but not HSP40 induced by bortezomib (Fig. 5f). Together, these data suggest that combining bortezomib with AMG9810 interferes with protective UPR activation, decreasing HSP70 and therefore sensitizing MM cells to bortezomib.

### Combining bortezomib with TRPV1 inhibitor AMG9810 modifies ubiquitin signaling pathway in MM cells

**Fig. 6:**
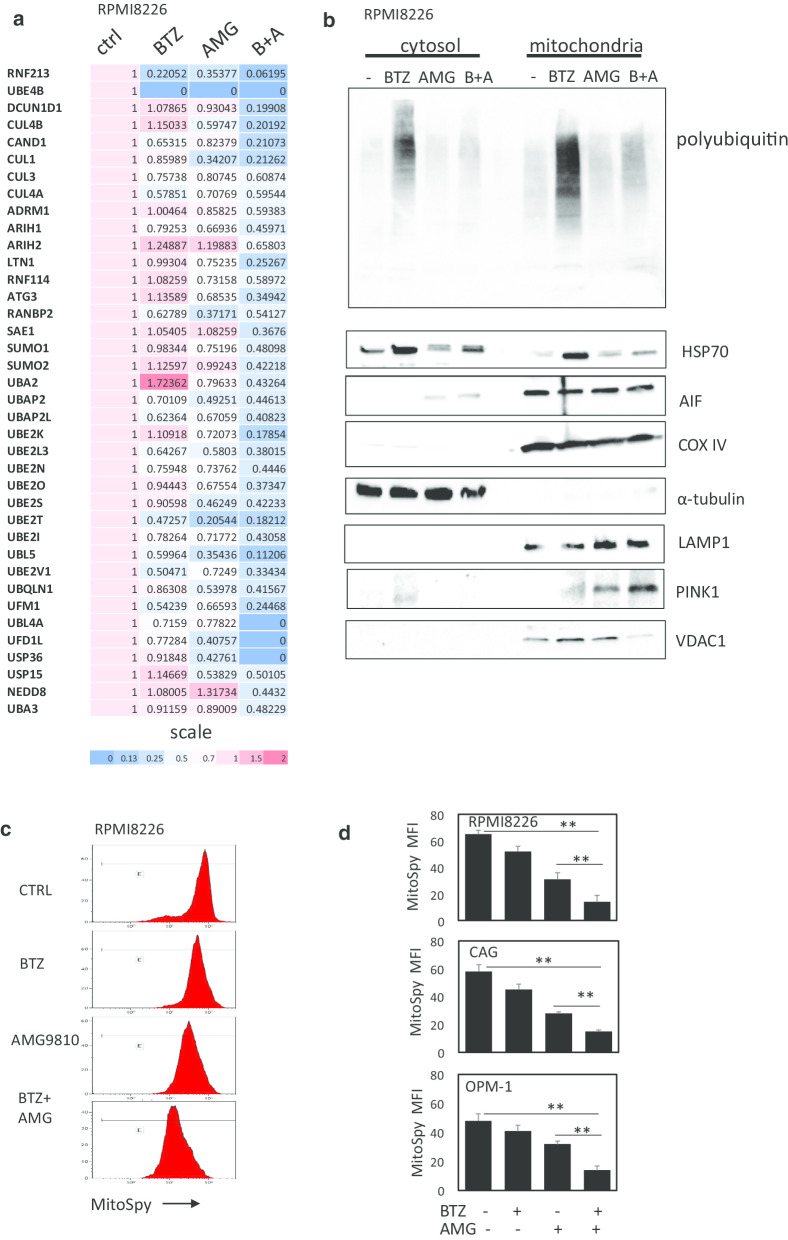
Combination of AMG810 with bortezomib suppresses ubiquitin signaling and induces mitophagy in MM cells. **a** RPMI8226 cells were exposed to bortezomib (5 nM), AMG9810 (10 µM) or their combination for 24 h and subjected to cell proteome analysis. **a** Heatmap of differentially expressed proteins associated with ubiquitin signaling pathway is presented (*p* < 0.05). **b** Western blot analysis of cytosolic and mitochondrial fractions of RPMI8226 cells that were exposed to bortezomib (5 nM), AMG9810 (10 µM) or their combination for 24 h. Blots were probed for poly-ubiquitin, HSP70, AIF, LAMP1, PINK1 and VDAC1. α-Tubulin and COX IV were used as markers for cytosolic and mitochondrial fractions, respectively. **c, d** Effect of bortezomib (5 nM), AMG9810 (10 µM) or their combination on mitochondrial mass in MM cells. Mitochondrial content was measured using MitoSpy staining and flow cytometry analysis. **c** Representative histograms showing the decrease in mitochondrial mass staining of RPMI8226 cells. **d** Graphs showing MitoSpy MFI levels in RPMI8226, CAG and OPM-1 cells presented as mean of triplicates ± SD (**p < 0.01)

To get further insight into the molecular mechanisms of bortezomib/AMG9810 synergism, the whole cell proteome profiles of the RPMI8226 cells treated with bortezomib, AMG9810 or their combination were compared using quantitative proteomic studies. Differentially expressed proteins were identified and quantified by LC–MS/MS mass spectrometry. The selection criteria for deregulation were the same for all the samples: identification based on at least two unique peptides and fold difference > 2.0 or < − 2.0. A total of 3343 non-redundant proteins were identified in both untreated and bortezomib/AMG9810-treated RPMI8226 cells. Proteomic analysis revealed unique molecular signature related to the modification of ubiquitin signaling pathway. Thus, 38 proteins related to the ubiquitylation machinery, including ubiquitin ligases and ubiquitin activating enzymes, were significantly downregulated upon combined bortezomib/AMG9810 treatment (Fig. 6a). In line with these results, AMG9810 abolished bortezomib-induced protein ubiquitination in MM cells (Additional file 1: Figure 4). Similarly, carfilzomib treatment promoted protein ubiquitination and increased HSP70 levels, while combination with AMG9810 effectively abrogated these carfilzomib-mediated effects, suggesting common mechanisms of AMG9810/proteasome inhibitors synergism in MM. Of note, TRPV1 agonist capsaicin had no effect on protein ubiquitination and HSP70 levels (Additional file 1: Fig. 5A), further emphasizing the specific and differential activity of TRPV1 antagonist AMG9810 in MM cells. Importantly, cells with over-expressed CXCR4 (RPMI8226-CXCR4) and bortezomib-resistant cells (RPMI8226-CXCR4-Bort^Res^) displayed elevated levels of protein ubiquitination in comparison with the original cell line RPMI8226, which was further increased upon bortezomib treatment. However, combination with AMG9810 impressively reduced the levels of ubiquitinated proteins in bortezomib-resistant MM cells (Fig. 5b).

### TRPV1 inhibition abolishes bortezomib-mediated mitochondrial UPR and promotes mitophagy in MM cells

Overload of misfolded proteins induced by proteasome inhibition may occur in mitochondria, initiating mitochondrial UPR (mtUPR) [35]. Indeed, the accumulation of ubiquitin-protein conjugates was detected in both cytosolic and mitochondrial fractions of bortezomib-treated RPMI8226 cells. However, the combination of bortezomib and AMG9810 reduced the levels of ubiquitinated proteins in both cytosol and mitochondria (Fig. 6b). Regulation of protein ubiquitination is known to be calcium dependent [36]. Therefore, TRPV1 inhibition and sustained perturbation in intracellular calcium may affect mitochondrial protein ubiquitination and homeostasis, inducing unresolved mitochondrial stress and increasing the sensitivity of MM cells to bortezomib. Mitochondrial HSP70 (mtHSP70) has been proposed as a potential marker for mtUPR, being indispensable for mitochondrial function, suppressing ROS-induced mitochondrial damage and permeabilization and protecting against cell death [37]. The latter was illustrated by an increase in mtHSP70 chaperon’s expression upon exposure to bortezomib. Importantly, AMG9810 treatment completely depleted bortezomib-induced HSP70 in the mitochondria. This was accompanied by release of mitochondrial pro-apoptotic proteins AIF to the cytosol, indicating extensive mitochondrial damage (Fig. 6b). Furthermore, lysosome-associated membrane protein 1 (LAMP1) levels in the mitochondrial fraction was increased upon AMG9810 treatment, suggesting lysosomal–mitochondrial interactions. Finally, we found that mitochondrial PTEN-induced putative kinase 1 (PINK1) levels were increased upon to exposure to AMG9810 alone or in combination with bortezomib. PINK1 stabilization on the outer membrane of impaired mitochondria is known to promote the selective lysosome-mediated clearance of defective mitochondria by mitophagy process [38]. In addition, the degradation of outer mitochondrial membrane protein VDAC was observed upon combined AMG9810/bortezomib treatment, further supporting the notion that TRPV1 inhibition in combination with bortezomib induces non-reversible mitochondrial damage and mitophagy (Fig. 6b). In line with the above, we detected significant decrease in mitochondrial mass in MM cells exposed to AMG9810/bortezomib treatment (Fig. 6c, d).

### Inhibition of ubiquitin pathway is essential for AMG9810-mediated effects in MM cells

To estimate the role of the ubiquitin pathway in AMG9810-mediated anti-MM effects, we utilized MLN4924 (pevonedistat), inhibitor of NEDD8 activating enzyme that suppresses Cullin-E3 ligase activity [39]. Among other ubiquitin-related proteins, we detected reduced NEDD8 levels in RPMI8226 cells upon bortezomib/AMG9810 exposure. Notably, combining MLN4924 with bortezomib significantly increased cell death in MM cells (Additional file 1: Figure 6A). Furthermore, NED88 inhibition using MLN4924 abolished bortezomib-induced ubiquitination and suppressed bortezomib-mediated protective mtUPR, as demonstrated by decrease in bortezomib-induced mtHSP70 levels (Additional file 1: Fig. 6B). Finally, NEDD8 inhibition in combination with bortezomib synergistically induced mitochondrial oxidative damage with subsequent MM cell death (Additional file 1: Fig. 6C, D). These results suggest that ubiquitin pathway and mitochondrial activity are critical targets of TRPV1 antagonist in MM cells.

### The combination of AMG9810 with bortezomib effectively targets MM cells in vivo in the BM niche

**Fig. 7:**
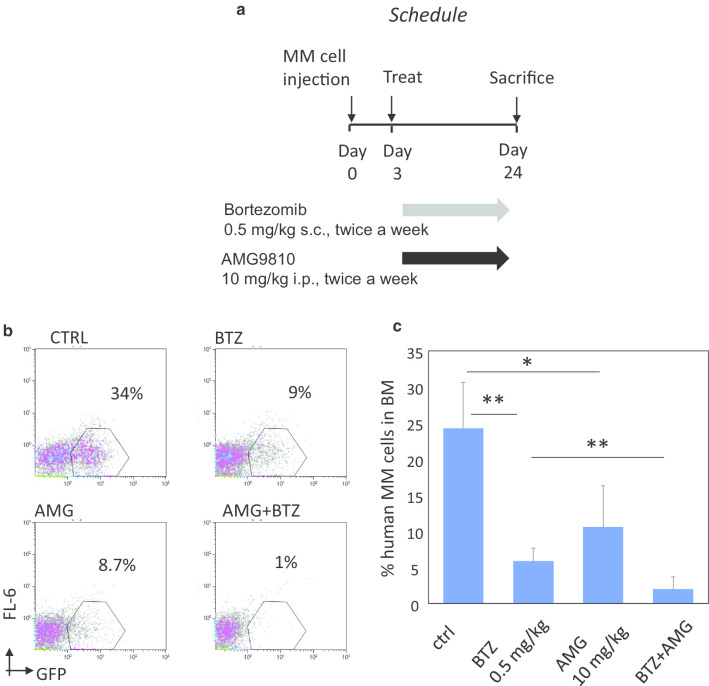
Combination of AMG9810 with bortezomib effectively targets MM tumor burden in vivo in the BM niche. **a** RPMI8226-CXCR4 (5 × 10^6^) were intravenously (i.v.) injected into NSG mice and three days later were treated with subcutaneous bortezomib injections (0.5 mg/kg) twice a week, total of 6 injections, intraperitoneal AMG9810 injections (10 mg/kg) twice a week, total of 6 injections or the combination of both agents. On day 24 following MM cell inoculation, mice were killed and the human MM burden in the murine BM was evaluated. **b**, **c** Response of tumor burden, presented as percent of RPMI8226-CXCR4 MM cells in the BM, five mice per group. Data are presented as mean ± SE, **p < 0.01

Lastly, we extended our in vitro findings and investigated the effectiveness of a combination approach consisting of AM9810 and bortezomib in vivo*,* using our xenograft model of MM with BM involvement. In this model intravenous injection of CXCR4-over-expressing myeloma cells into NSG mice results in preferential BM homing and development of a lethal disease resembling human MM [25]. Subsequently, NSG mice were inoculated with RPMI8226-CXCR4 cells and were treated with bortezomib (0.5 mg/kg), AMG9810 (10 mg/kg) or their combination for three weeks, total of 6 injections (Fig. 7a). As demonstrated in Fig. 7b, c, single agent AMG9810 treatment demonstrated marked anti-myeloma activity. Furthermore, the combination of both agents significantly reduced MM tumor load in the BM. Altogether, these results demonstrate the cooperative anti-myeloma activity of AMG9810 and bortezomib in vivo, enabling effective targeting of MM even in the protective BM microenvironment.

## Discussion

Despite the recent advances and improving the clinical outcomes of patients with myeloma, anti-MM treatment remains challenging and novel treatment strategies are urgently needed. Drug resistance remains a major challenge for MM cure.

Calcium is a critical secondary messenger that mediates various cellular processes in normal and cancer cells, keeping the balance between proliferation, differentiation and cell death [40–42]. Calcium is also one of the critical regulators of cell migration of various cell types, including tumor cells [40]. Furthermore, calcium signaling was proposed to play a role in drug resistance of cancer cells [43]. TRPV1 has been recognized as an important regulator of intracellular calcium levels and was shown to be functionally expressed in various cancer cells. These findings prompted us to consider the TRPV1 pathway as a therapeutic target in MM.

Our data highlight the novel role of TRPV1-dependent signaling in MM cell growth and drug sensitivity. TRPV1 inhibition using pharmacological inhibitor AMG9810 resulted in calcium-dependent accumulation of mitochondrial ROS, followed by mitochondrial destabilization and MM cell death. These results are in accordance with previous findings showing that calcium acts as a key regulator of mitochondrial function and that its overload can impair electron transport leading to ROS generation [26]. Furthermore, changes in mitochondrial calcium have been shown to regulate many cellular processes such as apoptosis [44], autophagy [45] and organelle crosstalk between mitochondria and ER [46]. Excessive calcium in the mitochondria is known to be associated with sustained activation of mitochondrial membrane permeability and the release of apoptotic factors such as cytochrome c into the cytosol [47]. Indeed, sustained activation of both apoptotic and autophagy pathways was observed upon TRPV1 inhibition in MM cells, including depletion of pro-apoptotic BCL-2 and MCL-1, increase in activated caspase 3 levels and increase in Annexin V staining. Additionally, increase in vesicle acidification and suppression of mTOR pathway suggested the activation of autophagy signaling following AMG9810 treatment. Furthermore, AMG9810-induced increase in CHOP indicated profound ER and mitochondrial stress mediated by TRPV1 inhibition.

Another important effect observed upon AMG9810 exposure was significant decrease in CXCR4 expression and function in MM cells, resulting in suppression of CXCL12-mediated signaling, while activation of TRPV1 using capsaicin enhanced CXCR4-mediated migration and adhesion. CXCR4/CXCL12 chemokine axis was shown to play pro-tumorigenic role in MM development and progression, inducing MM cell localization in the BM niche and promoting environment-mediated drug resistance [48]. CXCR4 activity is known to be dependent on calcium flux [49]. In normal and cancer cells, CXCL12 activation induces release of calcium from internal stores, triggering phospholipase C activation and the generation of inositol trisphosphate and diacylglycerol. Thus, calcium signaling is associated with processes that occur during metastasis, including cell migration and invasion [50]. Indeed, previous work demonstrated that increased extracellular calcium levels and intracellular calcium flux upregulated CXCR4 expression and activity in CD34+ hematopoietic stem cells [51]. In agreement with these findings, here we show that TRPV1 inhibition interferes with calcium signaling and suppresses both CXCR4 expression and activity in MM cells. On the contrary, TRPV1 activation using capsaicin promotes calcium influx, resulting in transient increase in cytosol calcium levels and, therefore, supporting CXCR4-mediated activity. Hence, our data demonstrate that TRPV1 is functionally connected to CXCR4-mediated activation, migration and adhesion of MM cells and reveal TRPV1 as a potential novel target in MM, possibly implicated in disease progression and refractoriness. Accordingly, inhibition of TRPV1 using AMG9810 effectively overcame stroma-mediated protection and restored the sensitivity of MM cells to bortezomib.

Moreover, combination of bortezomib with AMG9810 revealed synergistic anti-myeloma activity with unique mechanism. Simultaneous inhibition of proteasome and TRPV1 in MM cells promoted extensive mitochondrial damage with deleteriously increased mitochondrial ROS, induced lysosomal destabilization and uncompensated ER stress.

The major mechanism of bortezomib-induced cell death involves the accumulation of misfolded protein aggregates, which induce ER stress followed by an UPR. Activation of UPR may induce several lines of responses, acting to promote cellular survival or committing the cell to apoptosis. Increased expression of chaperons restores the folding capacity and ameliorate the stress, therefore reducing the responsiveness to bortezomib. Indeed, HSP70 induction is known to be a part of compensatory UPR in MM, while HSP70 inhibition was shown to be effective in combination with bortezomib [52]. Furthermore, the increased levels of chaperones including HSP70 have been associated with resistance to proteasome inhibitors in MM cells [53]. Our data show that TRPV1 inhibition using AMG9810 effectively downregulates HSP70 levels, overcoming the compensatory response induced by proteasome inhibitors bortezomib and carfilzomib. This may provide mechanistical hint, explaining the observed synergism between proteasome inhibitors and AMG9810 in MM cells. Noteworthy, the regulatory link between TRPV1 and HSP70 in epithelial cells was previously reported [54].

Notably, in addition to cytosolic HSP70, profound increase in mitochondrial HSP70 levels was detected upon bortezomib exposure, suggesting the induction of mitochondrial UPR. The mtHSP70 machinery plays a critical role in maintaining proteostasis balance in the mitochondria and is induced upon proteotoxic stress [55]. It was shown that elevated mtHSP70 prevents mitochondrial membrane depolarization and protects the cells from apoptosis by obliterating conformational changes in BAX and activating the AKT pathway [56]. Overexpression of mtHSP70 in cancer cells causes resistance against chemotherapeutic drugs like cisplatin [57], while reduction in mtHSP70 leads to increased apoptosis by decreasing mitochondrial membrane potential [58]. However, the role of mtUPR in responses to proteasome inhibitor therapy remains mainly unexplored. Thus, our work provides the evidence for mtHSP70 increase and mtUPR induction upon bortezomib treatment and suggests its involvement in cell protection and drug resistance. Importantly, AMG9810 completely depleted bortezomib-induced mtHSP70 and therefore abrogated protective mtUPR.

Another important finding of our study indicates that bortezomib-induced mtUPR involves the accumulation of ubiquitin-protein conjugates in mitochondrial fraction following bortezomib exposure. Inhibition of the degradation of ubiquitin-labeled proteins is known consequence of proteasome inhibitors. Of note, AMG9810 in combination with bortezomib significantly downregulated the levels of proteins related to ubiquitination system, including ubiquitin ligases and ubiquitin activating enzymes. Accordingly, TRPV1 inhibition with AMG9810 significantly diminished the accumulation of poly-ubiquitinated proteins in both the cytosol and the mitochondria. Inhibition of the ubiquitination system was shown to be effective in combination with proteasome inhibitors. For example, lenalidomide, an inhibitor of E3 ubiquitin ligase cereblon, is potent anti-MM drug [59]. Furthermore, ubiquitin-activating enzyme inhibition using TAK-243 molecule demonstrated anti-MM activity in preclinical models [60]. Recent evidences show a regulatory role for calcium in modulating ubiquitination system. Thus, ubiquitination activity of numerous E3 ligases is regulated by calcium [36]. Thereby, inhibition of ubiquitination by AMG9810 and potentiation of ER and mitochondrial stress by bortezomib can represent a mechanism that contribute to bortezomib/AMG9810 synergism. Furthermore, our data revealed increased basal level of protein ubiquitination in bortezomib-resistant cells, while AMG9810 in combination with bortezomib reduced the accumulation of ubiquitinated proteins in resistant cells. Therefore, modulation of the ubiquitination activity using TRPV1 inhibitor AMG9810 could be a new approach to target bortezomib-resistant MM cells.

Moreover, calcium is an important molecule of crosstalk at the ER-mitochondria junctions [46]. Accordingly, it was shown that E3 ligase Mahogunin Ring Finger 1(MGRN1) regulates in a calcium-dependent manner the exchanges between the ER and mitochondria and influences mitochondrial quality control through mitophagy [61]. Therefore, we hypothesize that TRPV1 inhibition results in sustained perturbation in intracellular calcium, interferes with ubiquitination activity and in combination with bortezomib promotes unresolved mitochondrial damage, inducing mitophagy in MM cells. This suggestion was further supported by our findings demonstrating the decrease in mitochondrial mass, mitochondrial-lysosomal fusion and mitochondrial accumulation of PINK1, a master regulator of UPR-induced mitophagy.

Finally, the effect of AMG9810 was validated in our in vivo model of CXCR4-driven human MM engrafting in murine BM. Our results demonstrate that single-agent treatment with AMG9810 targeted MM cells in the BM niche and significantly reduced tumor load. Most importantly, the combination of AMG9810 with bortezomib demonstrated preferential anti-MM activity, effectively reducing MM tumor load.

## Conclusions

Altogether, our data indicate that TRPV1 is implicated in MM cell survival, proliferation, migration, microenvironment interactions and stress response. TRPV1 inhibition by AMG9810 impairs calcium homeostasis, inhibits CXCR4-mediated migration and stromal protection, synergizes with bortezomib, targets ubiquitin pathway and cytoprotective mtUPR, impairs mitochondria, destabilizes lysosome and promotes MM cell death. These results unravel the mechanism mediating the strong synergistic anti-MM activity of bortezomib in combination with TRPV1 inhibition which may be translated into the clinic.

## Supplementary information

**Additional file 1**. Supplementary Methods and Results.

## Data Availability

All data generated or analyzed during this study are included in this published article and its Additional file 1 files.

## Abbreviations

AIF: Apoptosis-inducing factor
AO: Acridine orange
ATCC: American Type Culture Collection
BCL-2: B-cell lymphoma 2
BCL-XL: B-cell lymphoma extra large
BM: Bone marrow
BMSC: Bone marrow stromal cells
CFSE: Carboxyfluorescein succinimidyl ester
CHOP: C/EBP homologous protein
COX IV: Cytochrome c oxidase IV
CXCL12: C-X-C Motif Chemokine Ligand 12
CXCR4: C-X-C Motif Chemokine Receptor 4
DNA: Deoxyribonucleic acid
ECL: Enhanced luminol-based chemiluminescent
ELISA: Enzyme-bound immunosorbent analysis
ER: Endoplasmic reticulum
Erk 1/2: Extracellular signal-regulated protein kinase 1/2
FACS: Fluorescence-activated cell sorting
FCS: Fetal calf serum
FITC: Fluorescein isothiocyanate
GFP: Green fluorescent protein
HO-1: Heme oxygenase 1
HSP40: Heat-shock protein 40
HSP70: Heat-shock protein 70
qRT-PCR: Quantitative reverse transcription polymerase chain reaction
LAMP1: Lysosomal-associated membrane protein 1
MCL-1: Myeloid cell leukemia 1
MM: Multiple myeloma
mTOR: Mammalian target of rapamycin
PI: Propidium iodide
PINK1: PTEN-Induced Kinase 1
PVDF: Polyvinylidene fluoride
SDS-PAGE: Sodium dodecyl sulfate-polyacrylamide gel electrophoresis
RNA: Ribonucleic acid
ROS: Reactive oxygen species
UPR: Unfolded protein response
VDAC: Voltage-dependent anion channel
