# Supplementary material for: Blocking of Transient Receptor Potential Vanilloid 1 (TRPV1) promotes terminal mitophagy in multiple myeloma, disturbing calcium homeostasis and targeting ubiquitin pathway and bortezomib-induced unfolded protein response
Source: J Hematol Oncol. 2020 Nov 25;13:158. doi: 10.1186/s13045-020-00993-0 (PMC7687998; doi:10.1186/s13045-020-00993-0)
Supplement: Supplementary file 1 — Additional file 1. Supplementary Methods and Results. [file 13045_2020_993_MOESM1_ESM.docx]

**Additional file 1. Supplementary Methods and Results.**

**Additional Methods**

**XTT viability assay**

MM cells (2x10^4^ per 100 µl per well) were plated in 96-well flat plates in quadruplicate samples, with an increasing concentration of AMG9810 at various time points. Cell viability was assessed using the 2,3-bis(2-methoxy-4-nitro-5-sulfophenly)-5-[(phenylamino) carbonyl]-2H-tetrazolium hydroxide (XTT) assay (Biological Industries).

**Cell cycle analysis**

MM cells were exposed in vitro to increasing concentrations of bortezomib, AMG9810 or their combination. Cells were collected, washed with cold PBS, and fixed with 4% of paraformaldehyde (PFA) for 30 min. Fixed cells were re-suspended in staining buffer containing 0.1% saponin (Sigma-Aldrich) and 40 µg/ml RNase and incubated at 37^0^ C for 15 min. Cells were then stained with 10 µg/ml 7-amino-actinomycin D (7-AAD) (eBioscience) in dark for 30 min. DNA content was detected using FACS.

**Assessment of apoptosis**

Apoptosis was determined by staining with Annexin V-APC and PI according to the manufacturer’s instructions and analyzed by flow cytometry. The percentage of early apoptotic (Annexin V^+^/PI^-^) and late apoptotic/dead (Annexin V^+^/PI^+^) cells was quantified. Caspase 3 activity was evaluated by FACS analysis using the CaspGLOW Red Active Caspase-3 Staining Kit (BioVision) according to the manufacturer’s instructions.

**Cell adhesion assay**

MM cells (RPMI8226-EV and RPMI8226-CXCR4) were labeled with CFSE (5 µM), pre-treated with AMG9810 (5-10 µM) or capsaicin (10 µM) for 30 minutes in 37^0^ C and seeded over BM stromal cell monolayer in 24-well plates at a density of 2x10^5^ cells/ml in a medium supplemented with 1% FCS. Following 30 minutes of co-incubation in 37^0^ C, non-adherent cells were removed and the adherent cell fraction (including BMSCs and CFSE-labeled MM cells) was harvested with trypsin/EDTA. The cells were counted by FACS, CFSE-unlabeled BMSCs were gated out.

**RT-PCR analysis**

Total RNA from BM samples from patients with MM or from cultured MM cell lines was extracted using Trizol reagent (Invitrogen) according to the manufacturer’s instructions. To generate cDNA, 1 μg total RNA was reverse transcribed using the qScript cDNA Synthesis Kit (Quanta) according to the manufacturer's instructions. Real-time quantitative PCR (RT-qPCR) was performed in a final volume of 20 μL, containing 100 ng of total RNA-derived cDNAs, forward and reverse primers (300 nM) and PerfeCta SYBR Green FastMix (Quanta Biosciences), using the StepOnePlus Real Time PCR system (Applied Biosystems). Changes in expression levels were normalized to control β2-microglobulin using the ΔΔC_T_ method of relative quantification using the StepOne Software v2.2. Experiments were performed in triplicates for each sample. The sequences of primers are presented in Additional Table 1.

**Additional Table 1. Primers sequences.**

| Gene |  | Primer sequence |
| --- | --- | --- |
| human β2-microglobulin | Sense | AGGCTATCCAGCGTACTCCA |
|  | Antisense | TCAATGTCGGATGGATGAAA |
| human TRPV1 | Sense | GGCTGTCTTCATCATCCTGCTGCT |
|  | Antisense | GTTCTTGCTCTCCTGTGCGATCTTGT |
| human HO-1 | Sense | GCAGTCAGGCAGAGGGTGATAGAA |
|  | Antisense | GGGCTCTGGTCCTTGGTGTCAT |
| human CHOP | Sense | CCTGCAAGAGGTCCTGTCTT |
|  | Antisense | TCCTCCTCAGTCAGCCAAGC |
| human GADD34 | Sense | CACTTCAGGGGCTGGGGATA |
|  | Antisense | AGCGCACCTTTCTGGCCTTTA |
|  |  |  |
|  |  |  |

**Additional Figure 1. TRPV1 inhibition using AMG9810 induces dose-dependent apoptosis in MM cells.** MM cells and normal PBMCs were incubated with increasing doses of AMG9810 for 48 hours, apoptosis was measured using Annexin V/PI staining and FACS analysis. (A) Representative plots of RPMI8266 cells analyzed by FACS. (B) Graphs showing the percentages of apoptotic Annexin V+/ PI-positive RPMI8226, CAG, OPM-1 cells and normal PBMCs from representative experiment out of three repeats. Data are presented as mean of triplicates ±STDEV (**p<0.01).

**Additional figure 2.**

Using Chou-Talalay analyses, we examined the drugs interactions between bortezomib and AMG9810. RPMI8226, CAG and OPM-1 cells were treated with various concentrations of bortezomib (4 nM, 5 nM) and AMG9810 (5 µM, 10 µM) and their combination for 24 hours. Apoptosis data then utilized to calculate the CI by the Compusyn program, in which CI< 1 indicated synergistic interaction and CI=1 is additive.

**Additioanl figure 3. Differential effects of TRPV1 inhibitor AMG9810 and TRPV1 agonist capsaicin on** **mitochondrial calcium levels and mitochondrial ROS levels in MM cells.** (A) RPMI8226 cells were treated with AMG9810 (10 µM), or capsaicin (10 µM) for 30 min and 4 hours, and mitochondrial ROS levels were measured using MitoSOX and Annexin V staining. (B) MM cells were treated with AMG9810 (10 µM), or capsaicin (10 µM) for 24 hours, and cellular calcium levels were evaluated using eFluor514 staining and flow cytometry analysis. (C) MM cells were treated with AMG9810 (10 µM), capsaicin (10 µM), bortezomib (10 nM) or their combinations for 1 hour, and vesicle acidification was measured by flow cytometry using acridine orange (AO) dye. Data are presented as mean of triplicates ±STDEV (**p<0.01).


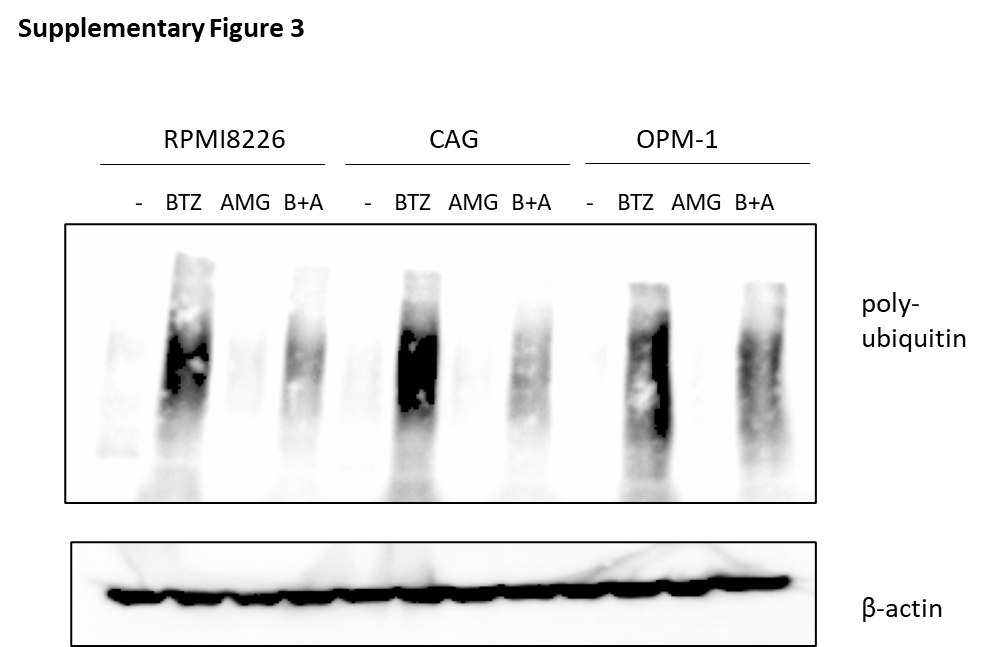


**Additional figure 4.** Western blot analysis of ubiquitinated proteins in total protein lysates of RPMI826, CAG and OPM-1 MM cells exposed to bortezomib (5 nM), AMG9810 (10 µM) or their combination for 24 hours. β-actin was used as internal control. Representative data from at least two independent experiments is shown.

**Additional figure 5.** (A) Western blot analysis of ubiquitinated proteins and HSP70 in total protein lysates of RPMI826 cells exposed to bortezomib (5 nM), carfilzomib (25 nM), AMG9810 (10 µM), capsaicin (10 µM) or drug combinations for 24 hours. (B) Western blot analysis of ubiquitinated proteins in total protein lysates of RPMI826-CXCR4 and RPMI8226-CXCR4-Bort ^Res^ cells exposed to bortezomib (10 nM), AMG9810 (10 µM) or drug combinations for 24 hours. β-actin was used as internal control. Representative data from two independent experiments is shown.

**Additioanl Figure 6**

**Additional figure 6. Effect of NEDD8 inhibitor MLN4924 on MM cell sensitivity to bortezomib.**

RPMI8226 and OPM-1 cells were exposed to bortezomib (5nM), MLN4924 (5 µM) or their combination for 24-48 hours. (A) Apoptosis was evaluated following 48 hours using Annexin V/PI staining and FACS analysis. (B) Western blot analysis of total protein lysates of RPMI8226 and OPM-1 cells that were exposed to bortezomib (5 nM), MLN4924 (5 µM) or their combination for 24 hours. Blots were probed for poly-ubiquitin and HSP70. β-actin was used as internal control. (C-D) Mitochondrial ROS levels and apoptosis were measured using MitoSOX and Annexin V staining.
